# Is the hysteroscopic surgery of isthmocele related to the niche dimensions?

**DOI:** 10.1590/1806-9282.20250047

**Published:** 2025-10-17

**Authors:** Sule Atalay Mert, Berna Dilbaz, Caner Kose, Tugba Kinay, Yaprak Engin-Ustun

**Affiliations:** 1 University of Health Sciences, Etlik Zubeyde Hanim Women's Health Training and Research Hospital, Department of Reproductive Endocrinology and Infertility – Ankara, Turkey.

**Keywords:** Hysteroscopy, Cesarean section, Niche, Ultrasonography

## Abstract

**OBJECTIVE:**

The aim of this study was to evaluate the impact of preoperative niche size on the success of hysteroscopic surgery in symptomatic isthmocele patients.

**METHODS:**

All patients who underwent hysteroscopic surgery for symptomatic isthmocele were recruited for this retrospective study. The dimensions of the niche, the apex of the niche to the uterine serosa (residual myometrial thickness), the depth of the wedge-shaped niche, and the width of the niche were measured before hysteroscopic repair of the isthmocele by ultrasonography. The present study assessed the correlation between preoperative niche dimensions and postoperative symptom resolution in a cohort of patients diagnosed with symptomatic isthmocele.

**RESULTS:**

Of the 29 patients evaluated, 72.4% (n=21) complained of postmenstrual spotting, while 27.5% (n=8) had both postmenstrual spotting and pelvic pain. Myometrial thickness was thinner in the postmenstrual spotting+pelvic pain group than in the postmenstrual spotting-only group (9.76±1.87 and 6.25±1.28, respectively, p≤0.001). After surgery, 75.8% of the patients were cured: 79.3% in the postmenstrual spotting-only group and 17.24% in the pelvic pain group were symptom-free. The residual myometrial thickness was greater both in the group that achieved total cure (9.50±2.02, p=0.002) and in the group with only postmenstrual spotting (9.39±2.04, p=0.005).

**CONCLUSION:**

This study demonstrated that patients with greater residual myometrial thickness had a higher success rate in hysteroscopic surgery for isthmocele-related symptoms. Preoperative niche measurements were considered a potential predictor of surgical outcomes.

## INTRODUCTION

Cesarean scar defects, called "isthmocele" or "niche," were first described by Poidevin in 1961^1^. During ultrasonography, a wedge-shaped anatomical defect in the uterine wall is observed as a hypoechoic area in the lower uterine segment at the site of the uterine cesarean incision^2^. The incidence of isthmocele diagnosed by transvaginal ultrasonography (TVUSG) is between 24 and 70%, and the rate increases from 56 to 84% with the use of sonohysterography^2–4^. The incidence of isthmocele increases with the increasing number of cesarean section (CS).

Isthmocele patients can be asymptomatic or may experience gynecological symptoms such as abnormal uterine bleeding (AUB), chronic pelvicpain, dyspareunia, secondary infertility, or obstetric complications^2,4–6^. The diagnostic criteria and a set protocol for the treatment of isthmocele are still missing from the guidelines. Conservative medical treatment with combined estrogen and progesterone therapy can be used for bleeding disorders, while hysteroscopy and laparoscopy, in combination, can be applied for surgical repair of the niche area^7^. In the literature, the large size of isthmocele is associated with symptoms such as postmenstrual spotting (PMS), intermenstrual spotting (IMS), dysmenorrhea, and chronic pelvic pain (PP)^6,8^. In the surgical treatment of isthmocele, hysteroscopic repair is recommended as the first choice if the residual myometrium thickness is at least 2.5–3 mm^9^.

Generally, studies in the literature compare surgical or medical treatment modalities and their pre- and postoperative results. In the present study, the relationship between preoperative ultrasound niche measurements and the presence and regression of symptoms in patients who underwent hysteroscopic metroplasty due to symptomatic isthmocele was evaluated.

## METHODS

This retrospective study was conducted at the Reproductive Endocrinology and Infertility Clinic, and patient data were extracted from the medical records. All consecutive patients who underwent hysteroscopic repair surgery for symptomatic isthmocele between May 2017 and January 2022 and met the inclusion criteria were included in the study. The study assessed the correlation between preoperative niche dimensions and postoperative symptom resolution in a cohort of patients diagnosed with symptomatic isthmocele. The study received approval from the local ethics committee (date: January 01, 2022 number: 01), and as a hospital policy, written signed consent was obtained from all patients for the anonymous use of medical data.

**Figure 1:**
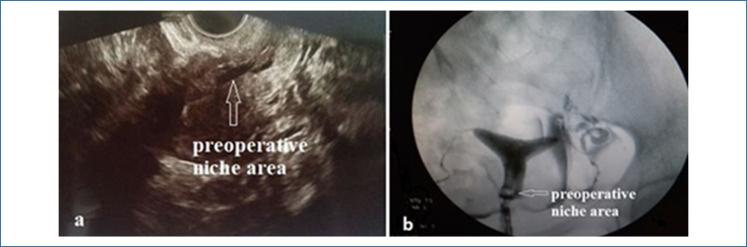
(a) Preoperative ultrasonographic image of the isthmocele niche, (b) Preoperative hysterosalpingographic image of the isthmocele niche.

Patients of reproductive age (18–45 years) who had undergone at least one cesarean delivery and presented with complaints of intermenstrual bleeding or pelvic pain, in whom a cesarean niche (isthmocele) was identified via two-dimensional (2D) and three-dimensional (3D) transvaginal ultrasonography (TVUSG) or hysterosalpingography (HSG), and who subsequently underwent hysteroscopic surgical repair due to this condition were included in the study (TVUSG; Figure 1a, HSG; Figure 1b).

The inclusion criteria were having a uterine niche at the cesarean scar site with AUB described as spotting or bleeding at the end of menstruation for more than 2 days (PMS), and/or intermittent or continuous chronic PP that persisted for 6 months or longer, independent of the menstrual cycle and sexual intercourse, after exclusion of endometriosis and adenomyosis. Furthermore, cases of secondary infertility were recorded in couples, even when standard infertility test results were within normal limits.

Exclusion criteria included peri- or postmenopausal status regardless of age (follicle-stimulating hormone [FSH] >15 IU/mL), suspected or confirmed pregnancy, a history of irregular menstrual cycles before CS, and known genital malignancy. Additionally, patients with concomitant uterine polyps, leiomyomas, or endometrial hyperplasia; endocrine disorders such as diabetes mellitus or thyroid dysfunction; and other conditions that may cause PP, such as endometriosis and adenomyosis detected by TVUSG, were also excluded. Furthermore, the study did not include patients who had undergone laparotomy and/or concomitant laparoscopy.

In the literature, there are various RMT cutoff values, such as 3 mm, 2.5 mm, and 3.5 mm, due to risks such as uterine perforation and bladder injury associated with hysteroscopic isthmocele surgical repair^10–12^. In our study, since we only evaluated patients who underwent hysteroscopy, patients with an RMT of 3 mm or less were excluded from the study in accordance with our clinical algorithm^9^.

### Assessment of the patients

**Figure 2:**
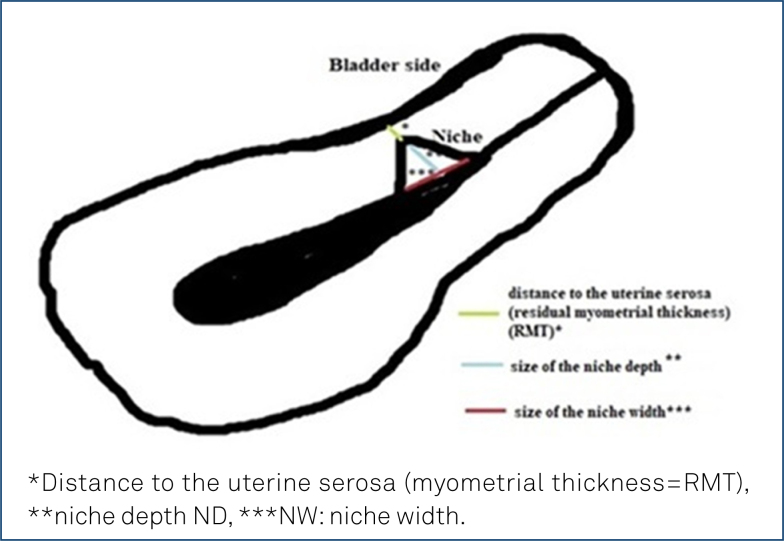
Niche measurements in patients with isthmocele.

All the patients were evaluated by the same team via TVUSG (Samsung H570A, 5–6 MHz) during the early follicular phase after routine gynecological examination. Ultrasonographic measurements of residual myometrial thickness (RMT), the depth of the wedge-shaped niche (ND), and the width of the niche (NW) (Figure 2) at the site of the previous CS scar were obtained by TVUSG without performing saline infusion sonography or sonohysterography.

### Operative procedures

All patients evaluated underwent hysteroscopy under regional (spinal) anesthesia. After adequate distension of the cavity was provided with 1.5% glycine, at an intrauterine pressure of 150 mmHg, the isthmocele site was resected by using monopolar energy with a loop resectoscope (Karl Storz, Germany). No medical treatment was administered routinely in the postoperative period.

### Follow-up

All patients were followed up for 1 year. Ultrasonographic measurements were not routinely repeated because the patients were living in other cities, and they came to the hospital for surgery only. Although postoperative follow-up of the patients was conducted via phone, patients’ symptoms and reproductive outcomes were recorded on follow-up forms.

### Sample size

The primary outcome of the study was the evaluation of symptom improvement after the surgery in patients with different isthmocele dimensions. Two symptoms were evaluated: spotting/bleeding and PP. For a study specifically addressing PP improvement, in the power analysis conducted with G-Power 3.0.0.1 software, when a 5% margin of error, 95% power, and a standard effect size of 1 were set, a minimum of eight patients are determined to be required^13^.

### Statistical analysis

Statistical analyses were performed using IBM SPSS 26.0 software (IBM Corporation, released 2019; IBM SPSS Statistics for Windows, Version 26.0; Armonk, NY, USA). The normality of the distribution of continuous data was checked by using the Kolmogorov-Smirnov test. Descriptive statistics were presented as mean±standard deviation or median (minimum–maximum). Categorical variables were presented as numbers and percentages. The independent samples t-test was used to analyze differences in normally distributed data. Data without a normal distribution were analyzed using the Mann-Whitney U test. A p<0.05 was considered statistically significant.

## RESULTS

A total of 29 patients who met the inclusion criteria were included, with a mean age of 33.0±4.19 years. Among them, 10 patients (34.5%) had accompanying complaints of secondary infertility, with a median duration of 19.80±7.51 months. More than half of the patients (57.5%, n=19) had only one previous CS.

When the patients’ complaints were evaluated, PMS was the most common complaint, with an incidence of 72.4% (21 out of 29), while PP+PMS was the second most common complaint, occurring in 27.6% (8 out of 29) of the patients (Table 1). When comparing the symptoms between obstetrical history parameters, no significant differences were found (Table 2). When the relationship between niche size and symptoms was evaluated, patients with both PMS and PP symptoms had thinner RMTs. However, no significant difference was found between symptoms and other measurements (ND and NW; Table 2).

**Table 1:** Clinical characteristics of the study population.

Characteristics		Mean±SD or median (min–max) or n (%)
	Age (year)	33.0±4.19
	Gravidity	2 (1–6)
	Parity	2 (1–3)
	Abortion	0 (0–4)
	Cesarean section number	1 (1–3)
	Secondary infertility	10 (34.5%)
	İnfertility duration (months)	19.80±7.51
**Symptoms**		
	PMS	21 (72.4%)
	PMS+PP	8 (27.6%)
**Ultrasonographic findings**		
	RMT (mm)	8.30±2.46
	ND (mm)	9.40±3.17
	NW (mm)	11.20±3.20
	Postoperative cure	22 (75.9%)

ND: niche depth; NW: width of the niche; PMS: postmenstrual spotting; PP: pelvic pain; RMT: residual myometrial thickness; SD: standard deviation. The data are presented as mean±SD or median (min–max). A t-test was applied for parametric values, and the Mann-Whitney U test was applied for nonparametric values.

**Table 2:** Clinical and ultrasonographic characteristics of the groups.

Total, n=29	PMS (n=21)	PMS+PP (n=8)	p-value
Age (year)	34.19±3.90	31.13±4.33	0.077
Gravidity	2 (1–6)	2 (1–4)	0.279
Parity	2 (1–3)	1 (1–3)	0.374
Abortion	0 (0–4)	0 (0–1)	0.943
Cesarean section number	1 (1–3)	1 (1–3)	0.830
RMT (mm)	9.76±1.87	6.25±1.28	<0.001*
ND (mm)	10 (5–16)	10.50 (9–15)	0.756
NW (mm)	10.57±3.49	13.25±2.44	0.057

ND: niche depth; NW: width of the niche; PMS: postmenstrual spotting; PP: pelvic pain; RMT: residual myometrial thickness. The data are presented as mean±SD or median (min–max). A t-test was applied for parametric values, and the Mann-Whitney U test was applied for nonparametric values.

* p<0.05 was considered statistically significant.

After hysteroscopic surgery, complete remission was achieved in 75.8% (22 out of 29) of the patients with PMS+PP symptoms. Among these patients, 79.3% (23 out of 29) experienced remission of PMS symptoms, while only 17.24% (5 out of 29) experienced remission of PP symptoms. In patients for whom remission was not achieved, the RMT was significantly lower in both the PMS+PP group and the PMS-only group (6.57±1.90, p=0.002, and 6.50±2.07, p=0.005, respectively, Table 3). Pregnancy was not achieved during the postoperative follow-up period in secondary infertility patients.

**Table 3:** Association between preoperative ultrasonographic measurements and postoperative outcomes.

M*	Cure (PMS+PP)			Improved PMS			Improved PP		
	Yes 75.8% (22/29)	No 24.13% (7/29)	p-value	Yes 79.3% (23/29)	No 20.6% (6/29)	p-value	Yes 17.24% (5/29)	No 10.3% 3 (3/29)	p-value
RMT (mm)	9.50±2.02	6.57±1.90	0.002	9.39±2.04	6.50±2.07	0.005	6 (5–8)	5 (5–7)	0.393
ND (mm)	10.5 (5–15)	10 (9–16)	0.354	10 (5–15)	10.5 (9–16)	0.302	14 (12–18)	12 (10–13)	0.143
NW (mm)	12 (5–16)	13 (10–18)	0.181	12 (5–16)	13 (10–18)	0.232	11 (9–15)	9 (9–10)	0.250

The data are presented as mean±SD or median (min–max). A t-test was applied for parametric values, the Mann-Whitney U test was applied for nonparametric values, and a p<0.05 indicates statistical significance.

M*: measurements; ND: niche depth; NW: width of the niche; PMS: postmenstrual spotting; PP: pelvic pain; RMT: residual myometrial thickness.

## DISCUSSION

Isthmocele repair surgery has gained importance because of the increase in CS rate in recent years. Patients may present with AUB symptoms such as PMS, IMS, menometrorrhagia, PP, unexplained secondary infertility, or a combination of these symptoms. In this study, complete regression of symptoms was observed in 75.8% (22 out of 29) of the patients with PMS+PP symptoms and in 79.3% of the patients with PMS symptoms. The RMT was lower in the PMS+PP group than in the PMS group.

Preoperative diagnosis of the isthmocele via sonohysterography is preferred, especially in patients with a myometrial wall thickness <2.5 mm. 3D TVUSG is becoming more widely used because it might lead to a better evaluation of the niche and its distance from the myometrial and serosal surfaces of the uterus than TVUSG^14^. When magnetic resonance imaging (MRI) and TVUSG were compared, there was no statistically significant difference despite the high positive predictive value of MRI^15^. In the present study, all the patients were evaluated with TVUSG, and niche measurements were carried out.

Thinner RMT can be interpreted as the presence of a deeper isthmocele that leads to increased blood collection inside the pouch. Blood collection in the pouch is explained to cause an inflammatory response^8,16^ and the release of inflammatory cytokines that affect the nerve fibers around the lesion. In a prospective study comparing pre- and postoperative symptoms in patients with isthmocele, AUB disappeared in 87.5% of patients within the first month, and in the second month after the operation, 96.8% of the patients achieved full recovery. Additionally, PP resolved 1 month after surgery in all patients^17^. In another study, after hysteroscopic surgery, the complaints of IMS and PP symptoms resolved in 80% of patients, while 13% had no improvement and 7% had some degree of improvement^18^. The high success rate achieved in resolving PMS (79.3%) aligns with broader findings on AUB management, where surgical correction of structural defects often leads to significant symptom improvement^6^. However, the persistence of PP in some patients suggests multifactorial etiologies beyond niche dimensions. In the present study, PMS was treated in 79.3% of women, and a total cure was observed in 75.8% of women with all the symptoms. The RMTs were thinner in these two groups, but the other dimensions (ND and NW) were not significantly different.

The hysteroscopic approach is the most widely used method as observed in published studies for the correction of isthmocele with adequate myometrial thickness at the isthmocele site, as well as for biopsy in cases of recurrent failure in infertile patients. Generally, hysteroscopy is a safe and successful diagnostic technique^19^. As it is a safe and effective surgical method, it can be repeated in certain cases. When hysteroscopic surgical resection was performed repeatedly for patients with ongoing symptoms, IMS complaints regressed, while no adverse effects on reproductive outcomes were observed^20^. No repeated hysteroscopy was performed in the patient groups in the present study.

A prospective cohort study evaluating the effectiveness of hysteroscopic resection of a uterine cesarean niche used bleeding symptoms and MRI measurements, reporting at least a 3-day reduction in abnormal bleeding. In contrast, our study showed improvement not only in bleeding but also in PP symptoms^21^.

Casadio et al. conducted a single-center, observational, prospective, cohort study involving 32 patients and reported a significant change in the mean visual analog scale (VAS) score for dyspareunia (-5.84; p<0.001), dysmenorrhea (-8.94; p<0.001), PP (-2.94; p<0.001), and AUB rates after channel-like (360°) hysteroscopic resection of the isthmocele (91 vs. 3%; p<0.001)^13^. In our study, dysmenorrhea and dyspareunia were not evaluated, and the success rate for PMS was 79.3%, with a total cure rate of 75.8%.

The present study demonstrated that ultrasonographic measurements of isthmocele size are useful in predicting symptom improvement after hysteroscopic surgery. Its findings may help guide patient selection for hysteroscopic surgery in clinical practice.

The major strength of this study is that it is the first research to report the association between isthmocele symptoms, surgical success, and ultrasound niche dimension measurements. However, there are also limitations to the study. One of the limitations is its retrospective design. The pre- and postoperative ultrasound measurements of niche sizes were obtained retrospectively from the participants’ medical records, which could introduce potential bias. Another limitation of the study is the small sample size. Prospective studies with larger patient populations are needed to support our results. Future prospective studies should incorporate standardized AUB classifications to better correlate niche anatomy with specific bleeding patterns^6^.

## CONCLUSION

The results of this study demonstrated that preoperative ultrasound isthmocele measurements could be a potential predictor of hysteroscopic surgery outcomes. In our study population, the mean RMT measurement was thicker in women with improved PMS symptoms after hysteroscopic surgery. However, there was no relationship between isthmocele measurements and PP symptoms. These findings may assist clinicians in selecting appropriate patients for hysteroscopic isthmocele surgery.

## Data Availability

The datasets generated and/or analyzed during the current study are available from the corresponding author upon reasonable request.
